# Immune Biomarkers in Triple-Negative Breast Cancer: Improving the Predictivity of Current Testing Methods

**DOI:** 10.3390/jpm13071176

**Published:** 2023-07-23

**Authors:** Francesca Maria Porta, Elham Sajjadi, Konstantinos Venetis, Chiara Frascarelli, Giulia Cursano, Elena Guerini-Rocco, Nicola Fusco, Mariia Ivanova

**Affiliations:** 1 Division of Pathology, IEO, European Institute of Oncology IRCCS, University of Milan, 20122 Milan, Italy; francescamaria.porta@ieo.it (F.M.P.); elham.sajjadi@ieo.it (E.S.); konstantinos.venetis@ieo.it (K.V.); chiara.frascarelli@ieo.it (C.F.); giulia.cursano@ieo.it (G.C.); elena.guerinirocco@ieo.it (E.G.-R.); mariia.ivanova@ieo.it (M.I.); 2 Department of Oncology and Hemato-Oncology, University of Milan, 20122 Milan, Italy

**Keywords:** breast cancer, TNBC, biomarkers, immune landscape, PD-L1, tumor-infiltrating lymphocytes, mismatch repair, microsatellite instability, artificial intelligence

## Abstract

Triple-negative breast cancer (TNBC) poses a significant challenge in terms of prognosis and disease recurrence. The limited treatment options and the development of resistance to chemotherapy make it particularly difficult to manage these patients. However, recent research has been shifting its focus towards biomarker-based approaches for TNBC, with a particular emphasis on the tumor immune landscape. Immune biomarkers in TNBC are now a subject of great interest due to the presence of tumor-infiltrating lymphocytes (TILs) in these tumors. This characteristic often coincides with the presence of PD-L1 expression on both neoplastic cells and immune cells within the tumor microenvironment. Furthermore, a subset of TNBC harbor mismatch repair deficient (dMMR) TNBC, which is frequently accompanied by microsatellite instability (MSI). All of these immune biomarkers hold actionable potential for guiding patient selection in immunotherapy. To fully capitalize on these opportunities, the identification of additional or complementary biomarkers and the implementation of highly customized testing strategies are of paramount importance in TNBC. In this regard, this article aims to provide an overview of the current state of the art in immune-related biomarkers for TNBC. Specifically, it focuses on the various testing methodologies available and sheds light on the immediate future perspectives for patient selection. By delving into the advancements made in understanding the immune landscape of TNBC, this study aims to contribute to the growing body of knowledge in the field. The ultimate goal is to pave the way for the development of more personalized testing strategies, ultimately improving outcomes for TNBC patients.

## 1. Introduction: Immune Actionability of TNBC

Triple-negative breast cancer (TNBC) represents a highly aggressive and heterogeneous subtype of breast cancer (BC), characterized by the absence of estrogen receptor (ER), progesterone receptor (PgR), and HER2 expression [1,2]. The management of these patients poses significant challenges, primarily due to the scarcity of effective treatment choices and the development of resistance to chemotherapy [2,3]. However, an avenue of hope lies in immunotherapy, harnessing the remarkable adaptability of the immune system, particularly in the context of TNBC, which exhibits the highest level of immunogenicity among breast-cancer subtypes [4,5]. In particular, immune-checkpoint inhibitors (ICIs) have broadened the treatment landscape of TNBC, both in the neoadjuvant and adjuvant settings [6]. This type of immunotherapy in metastatic TNBC (mTNBC) is biomarker-based [7]. Numerous immune-related biomarkers are currently approved in the clinical management of mTNBC, such as programmed death-ligand 1 (PD-L1), tumor-infiltrating lymphocytes (TILs), and mismatch repair (MMR) system [8,9,10]. Accurate pathological testing holds utmost significance in the assessment of patients with these biomarkers; however, testing strategies may differ depending on the specific diagnostic scenario, encompassing sample availability and diagnostic assays. The key to improving the outcome of TNBC lies in the harmonization and complementation of current testing strategies for actionable biomarkers. Here, we offer an updated overview of the present advancements in immune-related biomarkers for TNBC to identify personalized testing strategies.

## 2. PD-L1

The assessment of PD-L1 has become a routine clinical practice to predict the efficacy of ICIs in mTNBC [11,12]. Depending on the assay and scoring system used, the prevalence of PD-L1 positivity in TNBC varies between 17% and 59% [13]. Two immunotherapy compounds are currently approved for PD-L1-positive TNBC, namely, atezolizumab (an anti-PD-L1 monoclonal antibody) and pembrolizumab (an anti-PD-1). The gold standard for the evaluation of PD-L1 expression consists of immunohistochemical (IHC) staining on formalin-fixed paraffin-embedded (FFPE) sections [14]. Research conducted on mTNBC has demonstrated that the expression of PD-L1 may be observed on either tumor cells (TCs) or TILs [15,16,17]. Depending on the specific assay used for the test, different cellular compartments should be evaluated for PD-L1 expression. For this reason, various types of PD-L1 tests are available, accompanied by different scoring systems, each having distinct threshold values that determine patient eligibility for specific drugs [7,18,19,20]. In particular, a combined positive score (CPS) cut-off value of 10 is used to identify patients who are suitable for pembrolizumab treatment, while a 1% immune-cell (IC) cut-off value guides the selection for atezolizumab treatment [2,10,21,22]. There are currently four FDA-registered PD-L1 IHC assays that employ four distinct PD-L1 antibodies (22C3, 28–8, SP263, SP142) and are implemented on two different IHC platforms (i.e., Dako and Ventana). Moreover, these assays use four different scoring systems with distinct threshold values, determining the eligibility of patients for different medications [17,19,23,24,25]. The presence of multiple assays requiring diverse platforms can pose considerable technical hurdles for numerous laboratories, thereby introducing the potential for interlaboratory variability in the obtained results. Furthermore, the utilization of multiple scoring systems demands specialized training to minimize interobserver variability among pathologists. [26].

### 2.1. IC Score

Immune cell score (IC) is used to evaluate PD-L1 expression using the VENTANA PD-L1 (SP142) assay [17,27]. It is defined as the proportion of tumor area (the area occupied by viable tumor cells and their associated intra- and peritumoral stroma) occupied by PD-L1 stained ICs [28]. All immune cells (lymphocytes, macrophages, dendritic cells, and granulocytes) exhibiting any degree of staining, regardless of type and intensity, are considered positive for PD-L1 expression [15,29]. Tumor-cell staining may be observed, but should be disregarded. The cut-off value, making the TNBC patient eligible for atezolizumab therapy, is IC ≥ 1% [26]. From the results of the IMpassion-130 trial (NCT02425891) [11], atezolizumab has been recommended as a first-line therapy option for PD-L1-positive TNBC, either de novo or after at least 12 months since (neo)adjuvant chemotherapy, using the companion test of SP142 PD-L1 immunohistochemical assay [12]. Subsequently, using the same assay, atezolizumab plus nab-paclitaxel therapy has been approved for locally advanced breast cancer or mTNBC in cases where the tumor-associated immune cells express PD-L1 with the IC score ≥ 1% [29]. Assessment of PD-L1 expression via IC score has been demonstrated to have high validity and reproducibility when performed through the provided diagnostic kit and strictly following the manufacturer’s operating procedures and by specifically trained pathologists [30]. However, its use may present certain difficulties when evaluating suboptimal material. It could be challenging to correctly identify tumor area and peritumoral stroma on fragmented tissue samples or small biopsies. Previously, it has been highlighted that PD-L1 status assessments on biopsy specimens may not provide an accurate representation of the immunologic landscape and may differ from a surgical specimen’s results, thus depriving the patient of a treatment option [31,32]. Overall, the expression of PD-L1 in ICs has been found to be significantly higher in primary tumors compared to metastatic ones [33]. This is a common instance in routine clinical practice, where patients eligible for ICI therapy often present with advanced-stage disease and multiple comorbidities, making them unsuitable for the invasive procedures required to obtain larger tumor samples. In addition, this assay has been validated for use on lymph node metastases, where intratumoral ICs may not be easily distinguishable from the resident immune population [33,34]. Another weakness of this scoring algorithm is its sole reliance on IC positivity. It has been observed that interpathologist concordance in IC evaluation is lower with respect to tumor cell score (TC) [35,36]. This could be explained by the intrinsic difficulty in identifying ICs such as macrophages or dendritic cells within areas of closely packed tumor cells on both hematoxylin and eosin and IHC slides [14]. These data have been supported by the results of the Impassion130 study, showing that stromal TIL levels are synergistic with PD-L1 expression [37].

### 2.2. CPS

Combined positive score (CPS) is the scoring system developed for the 22C3 (Dako pharmDx) IHC assay. It is implemented to select patients who could benefit from pembrolizumab therapy [12,38]. CPS is calculated by summing the number of PD-L1 stained TCs and a subset of ICs (lymphocytes and macrophages) and dividing the result by the total number of viable TCs, multiplied by 100. Even though its value could theoretically exceed 100, CPS is expressed on a scale from 0 to 100 [12,26]. In TNBC, PD-L1 IHC 22C3 pharmDx is an FDA-approved diagnostic test for pembrolizumab treatment (in CPS ≥ 10) based on the results of the KEYNOTE-355 study [39,40,41]. One of the points of strength of this scoring system is that it takes into account both TCs and ICs, providing a more comprehensive assessment of the tumor microenvironment in relation to ICI status. In addition, since this assay requires only the quantitative assessment of cells and not the definition of a tumor area, it may also be used on very small or fragmented tissue samples as well as cytological samples, provided that a minimum of 100 cells can be evaluated [38]. Based on the meta-analysis of 57 studies on the accuracy of laboratory-developed PD-L1 tests, the 22C3 tests achieved the best results, with both sensitivity and specificity of 100% in eight out of nine studies [39], with good pathologists’ interobserver reproducibility [42,43]. The challenges of CPS scoring may be evoked by the spatial heterogeneity of PD-L1, potentially leading to missed scoring due to poorly circumscribed cells, misinterpretation of cytoplasmic/background staining or endogenous pigments, making the asessment of borderline cases challenging [42,44]. Some clones used for CPS (such as 28-8, 22C3) may demonstrate weak cytoplasmic staining of the tumor cells, which should be disregarded, as only membrane staining is taken into account [26]. The presence of a dense inflammatory infiltrate surrounding tumors may also complicate the assessment of cell proportions [44]. Nevertheless, Beckers et al. have demonstrated that PD-L1 expression in ICs may reflect an association with tumor-infiltrating-lymphocyte (TIL)-mediated antitumor inflammatory responses, even if not independently prognostic in TNBC alone [45,46].

### 2.3. Harmonization of PD-L1 Test

Attempts at harmonization of PD-L1 IHC antibodies and staining platforms are ongoing [23] While PD-L1 IHC can be used to predict the likelihood of response to anti-PD-1 or anti-PD-L1 therapy, a subset of patients who test negative for PD-L1 expression may still exhibit a favorable response to these treatments [47]. The comparative evaluation of CPS and IC scoring systems has generated conflicting findings and inadequate agreement across multiple studies [17,25,48,49,50]. These results indicate that different PD-L1 assays cannot be considered interchangeable or equivalent in the selection of patients affected by mTNBC who would benefit from therapy. Therefore, a careful selection of an appropriate assay is essential for effective treatment, considering the potential toxicity associated with combination therapies [49,51]. Standardized guidelines and thresholds for interpreting PD-L1 scores vary across different tumor types and specific assays used for evaluation. It is crucial to follow the specific guidelines provided by regulatory agencies, or professional organizations, to ensure consistent and accurate interpretation of PD-L1 in clinical practice [52]. Given these challenges, the identification of additional alternative biomarkers assumes critical importance in refining the selection of patients who are most likely to respond positively to specific therapies. By exploring and incorporating additional biomarkers, the precision and accuracy of patient stratification can be improved, thus enabling the administration of personalized treatments that optimize therapeutic outcomes.

The comparison of immunohistochemical companion diagnostic assays for PD-L1 assessment in TNBC are given in Table 1.

## 3. Tumor-Infiltrating Lymphocytes (TILs)

Tumor-infiltrating lymphocytes (TILs) comprise a heterogeneous group of immune cells, predominantly consisting of T cells, along with smaller proportions of B cells and NK cells, playing an important part in the TME [53]. The presence of TILs holds favorable prognostic significance for TNBC [54,55]. Research demonstrates, that targeting the regulation of immune checkpoints between tumor cells and T lymphocytes has the potential to enhance the prognosis of TNBC [56]. A high TIL count has been linked to favorable outcomes in terms of disease-free survival (DFS), OS, and response to neoadjuvant chemotherapy [56,57].

### 3.1. TIL Evaluation

Although the significance of TILs is widely acknowledged, there are certain challenges associated with their assessment, particularly for patients with recurrent breast cancer. Typically, TILs are evaluated in the primary tumor, as obtaining biopsies from recurrent lesions can be problematic [37]. Even if a biopsy from a recurrent site is obtained and evaluated, it remains uncertain whether it can be interpreted in the same manner as the primary lesion due to potential variations in the degree of TIL infiltration and the immune-cell profile concerning the metastatic organ [37]. Consequently, there is a pressing need to establish simple approaches for assessing the real-time antitumor immune response in patients with recurrent disease, such as liquid biopsies through peripheral blood sampling [58]. In routine practice, TILs are evaluated using hematoxylin-and-eosin (H&E)-stained tissue slides and classified into stromal TILs (sTILs) and intratumoral TILs (iTILs) [59]. According to original guidelines presented by Denkert et al. [60], the scoring of TILs should be performed exclusively based on the percentage of stromal areas, excluding the areas occupied by carcinoma cells from the total surface area assessed to avoid tumor growth pattern having an impact on the final score. Furthermore, scoring of TILs in regions affected by crush artifacts, necrosis, inflammation around biopsy sites, or extensive central regressive hyalinization should be avoided [60]. Biopsies displaying extent necrosis are deemed unsuitable for TIL assessments and should not be scored [60]. To enhance the consistency of TIL assessments and facilitate their integration and interpretation in clinical trials, the International Immuno-Oncology Biomarker Working Group on Breast Cancer has formulated guidelines for standardized TIL scoring in BC [61]. The level of detail provided in the methodology is comprehensive enough to establish a consistent and practical foundation for TIL assessments in future studies [62].

### 3.2. TILs in Breast Cancer

There is a differential immune microenvironment in advanced- compared with early stage TNBC. TILs have been detected in the stroma of up to 60% of BC cases, with the highest frequencies in HER2+ and TNBC [63]. The tumor-infiltrating lymphocyte quantity is significantly lower in metastatic disease compared with primary tumors, demonstrating prognostic significance and its potential predictive value [62,64]. Recent clinical trials have indicated a correlation between TILs and the response to multiple treatment modalities, including both cytotoxic and immune therapies, with a particular emphasis on patients with TNBC, due to their complex clinical management [63]. Emerging evidence provides support for the clinical relevance of TILs in predicting favorable responses to immunotherapy in both early and advanced TNBC cases [63]. In early BC, the presence of higher stromal TIL levels has been associated with a better response to the ICIs in numerous trials, indicating that fluctuation of TIL levels over time holds promise as a potential indicator for predicting the efficacy of ICI treatment [65,66]. In the context of metastatic spread, elevated TIL levels have been associated with an increased probability of positive response to ICI treatment, as well as improved OS and DFS in patients with TNBC [53,67,68,69]. In locally advanced TNBC, a high TIL count has demonstrated noteworthy predictive and prognostic implications. Patients with high TIL counts exhibit a remarkable response rate of 88% to neoadjuvant chemotherapy, whereas those with low TIL counts display a significantly diminished response [70]. The phase III IMpassion130 trial demonstrated that the combination of atezolizumab and nab-paclitaxel resulted in a longer PFS in TNBC patients with a TIL level of 10% or higher [37]. When TIL levels were combined with PD-L1 positivity, the difference in PFS became even more significant, indicating a stronger therapeutic effect (HR: 0.54, 95% CI = 0.39–0.75) [37]. Immunotherapy shows great promise as a viable strategy for TNBC due to its relatively higher levels of TILs and PD-L1 expression compared to other BC subtypes [71]. However, considering the retrospective nature of most available data, additional independent prospective studies are necessary to validate these findings. Of note, PD-L1 expression and TIL levels have been found to strongly correlate with each other, suggesting a surrogate of the activated host antitumor immune response [72]. Interestingly, some research provides evidence that both PD-L1 expression and TILs in TNBC are associated with better patient outcomes [45]. However, it is worth noting that high TIL levels alone have been an independent prognostic factor for breast-cancer-specific mortality [45]. Specific cell subsets composition of different TILs has been shown to impact the response to ICIs as well, where regulatory T cells seem to be a key immunosuppressive player in the TME [66,73]. A study with a continuous evaluation through baseline and on-treatment biopsies revealed that advanced TNBC patients who responded positively to nivolumab monotherapy following an immune induction phase exhibited higher levels of TILs and CD8+ T lymphocytes compared to nonresponders [74]. The prognostic value of different TIL subsets has been further explored in a study of 259 stage I–III TNBC patients, demonstrating that additionally to stromal TILs, total and intratumoral CD8+ T cells are the independent prognostic factors for DFS [75]. Detailed analyses of TIL compositions have revealed a correlation between the presence of CD8+ T cells and T regulatory cells with improved treatment outcomes in chemoimmunotherapy, as observed in various trials [74,76,77]. Notably, single-cell data have suggested a potential connection between tertiary lymphoid structures, characterized by the aggregation of lymphoid cells and the colocalization of CXCL13+ CD8+ T cells, CD4+ T cells, and CXCR5+ B cells, with the response to ICI [53]. Overall, these data indicate that the contribution of different immune-cell subsets in TME may aid to refine the prognostic model for TNBCs, where in-depth genetic studies are likely warranted and underway. A study of nine treatment-naïve TNBCs has subjected the patients to next-generation sequencing (NGS), subdividing them into TIL-high and -low groups [78]. There was no significant difference in gene expression between patient groups, except Phosphatase and Tensin Homolog (PTEN) loss in the TIL-high group simultaneously with high PD-L1 levels, suggesting PTEN loss and high expression of PD-L1 in TIL-high TNBC to be a biomarker for ICI therapy [78]. Future research endeavors aiming to uncover the clinical actionability of TILs in BC, with a particular focus on TNBC, rely on achieving a comprehensive understanding of the breast cancer pathology specifics and the significance of TIL subtyping. Considering the cost-effectiveness and robust prognostic value, TILs may become a reliable biomarker to be used in antitumor T-cell-mediated immunity assessments. Given the complex nature of clinical management in TNBC, the integration of TILs into routine clinical practice should be seriously contemplated. Furthermore, ongoing investigations on combined immunotherapy present a promising evolution towards a tailored approach to this particular patient subgroup. The overview of major clinical trials assessing immunotherapy-specific biomarkers in TNBC is represented in Table 2.

## 4. Mismatch Repair System (MMR)

The mismatch-repair (MMR) system serves as a natural defense mechanism against DNA base mispairing, playing a vital role in human physiology [79]. This intricate cellular process is influenced by both external factors and internal mechanisms, working together to maintain the integrity of DNA [80]. Disruptions in the MMR complex can lead to genome instability, creating a favorable environment for the development of cancer [81]. In recent years, the clinical significance of MMR alterations in TNBC has gained significant attention [82]. It not only aids in the identification of inherited conditions, but also plays a crucial role in patient prognosis, predicting the effectiveness of ICIs, and early detection of therapy resistance [83]. The presence of MMR deficiency is exceptionally rare in breast cancer, accounting for only 1 to 2% of cases, and around 6% in TNBCs [84]. The evaluation of this condition can be accomplished through the analysis of MMR protein expression or by identifying microsatellite instability (MSI), which serves as an observable outcome of deficient mismatch repair (dMMR) [71,85].

The diagnosis of dMMR is typically performed through IHC on FFPE tissue sections, where the loss of nuclear immunostaining for at least one of the four routinely examined MMR proteins (MSH2, MSH6, MLH1, and PMS2) is observed. The available methods for detecting MSI include polymerase chain reaction (PCR), multiplex PCR, and next-generation sequencing (NGS). Typically, for these tests, DNA is extracted from both tumor and normal control FFPE tissue samples [85]. The presence of high-frequency microsatellite instability (MSI-H) in tumors triggers specific immune responses mediated by TILs, which possess antitumor properties. As a result, MSI-H/dMMR tumors have a higher likelihood of responding to ICI therapy [51]. Based on this evidence, the FDA approval of pembrolizumab includes all solid tumors that exhibit this specific genetic alteration [86]. Consequently, there is are increasing recommendations for the routine oncological care of patients with solid tumors to include universal screening for MMR and MSI status, regardless of the cancer’s origin [87]. The reported low percentage of dMMR breast-cancer cases may be attributed to the lack of companion diagnostics assays (CDx) and/or specific guidelines for MMR analysis in breast tumors, as well as the utilization of different testing methods, such as the direct sequencing of microsatellite markers, NGS, and IHC for the four MMR proteins. In breast cancer, the loss of MMR proteins is more frequently detected compared to MSI [87,88]. Therefore, it is important to note that IHC for MMR proteins and MSI testing cannot be used interchangeably, unlike in other tumor types [88]. One of the biggest studies involved 1084 breast-invasive-carcinoma cases from the Breast Invasive Carcinoma dataset of The Cancer Genome Atlas (TCGA) PanCancer Atlas, spanning samples using a multisample protein–protein interaction analysis tool and utilizing the RNA-sequencing data from cBioPortal in treatment-naïve patients [89]. Authors have found the highest prevalence of dMMR in the TNBC group, which also correlated with improved patient survival [89]. Owing to its rarity, MMR/MSI testing has been ambiguously discussed as an ICI biomarker in TNBC [71]. Nevertheless, the predictive value of MMR deficiency has been demonstrated in two separate studies that examined the response of mTNBC and luminal BC patients to nivolumab and pembrolizumab, respectively [71]. More recently, MMR testing has emerged as a molecular target in precision oncology for TNBC, as it has been observed to exhibit high sensitivity to immunotherapy [90]. The infrequent occurrence of MMR and MSI alterations in BC raises several questions regarding the most effective testing strategy. To enhance the understanding of MMR deficiency and provide more therapeutic options for TNBC patients, it has been studied in association with other biomarkers. The study of forty-four TNBC patients revealed four (11%) dMMR cases, three of which were PD-L1 negative and harbored high TILs [91]. These findings give rise to the suggestion that the adoption of multiple-biomarker testing (e.g., PD-L1, TILs, and MMR) may improve TNBC patients’ selection for immunotherapy eligibility [91]. On the contrary, some research data have shown no association of MMR/MSI status with PD-L1 expression in TNBC [87]. Furthermore, the study of 145 TIL-high TNBCs demonstrated the low MSI-H prevalence in this cohort, suggesting there might be no specific distribution pattern of MSI-H tumors across breast cancers at all [92]. Within this framework, the assessment of PTEN expression, a critical tumor suppressor that regulates cell growth, proliferation, and survival, and is also implicated in the MMR system and overall DNA damage response, has been proposed as a method to identify MMR-proficient (pMMR) breast tumors. Its somatic mutations were previously found more prevalent in BCs associated with MMR variant carriers, which mostly result in dMMR and have an unfavorable prognosis [93]. However, by now, the adoption of this approach has certain limitations and warrants approval [71]. One of the biggest published studies on BC aimed to identify mutational signatures in a whole-genome-sequencing (WGS) dataset consisting of 640 patients. The objective was to identify dMMR-deficient breast tumors [94]. Given that WGS may directly reflect the disruption of the MMR pathway, the authors assume it may potentially surpass existing biomarkers for detecting MMR deficiency, offering a greater level of sensitivity, which is of utmost importance, especially in dMMR-rare tumor types [94]. Although the discrepancy between MMR IHC and MSI has been demonstrated to be substantial [82], the validation of IHC and its relationship to NGS results will be important for guiding diagnostic MMR workflows in BC [89]. The molecular subtyping of the tumor-based MMR status holds significance in characterizing tumors as dMMR, thereby guiding the selection of ICIs and other targeted therapies for TNBC and BC overall [89].

## 5. Role of AI to Complement Immune-Biomarker Testing

**Figure 1:**
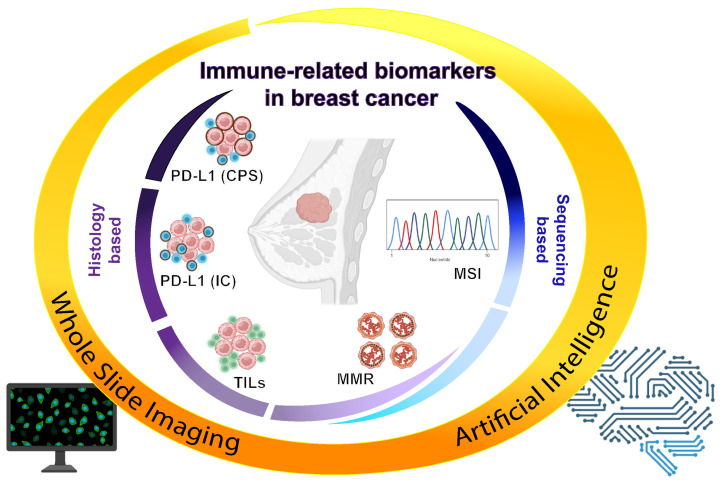
Overview and applications of digital and computational pathology to enhance testing of immune-related biomarkers in TNBC. PD-L1 (CPS): programmed death-1 ligand (combined positive score); PD-L1 (IC): programmed death-1 ligand (immune cell score); TILs: tumor-infiltrating lymphocytes; MMR: mismatch repair; MSI: microsatellite instability.

To ensure optimal therapeutic care for patients, clinically actionable biomarkers for TNBC must be accurately and consistently characterized. However, there may be issues with the standard methods (as IHC, RT-PCR, and NGS) for analyzing these biomarkers, such as variability and repeatability between observers and platforms [95]. Effective remedies continue to be subtle due to the extremely complex nature of this crucial task in pathology [96,97]. From another point, the type and quantity of specimens to be tested are continuously changing in breast cancer due to the widespread use of minimally invasive/noninvasive techniques [96]. Conversely, there is an increasing need to undertake a more thorough investigation using a wider range of biomarkers in this multifaceted situation. Even the use of theoretically more objective molecular-analysis techniques is usually complicated by a number of issues [97]. Artificial intelligence (AI) has recently been offered as a new possible tool to support diagnostic algorithms in clinical practice. Machine learning (ML), deep learning (DL), and convolutional neural networks (CNNs) are a few of the many subsets or approaches of AI that may be used to extract and evaluate data [98]. The application of these tools in digital pathology could enable the mining of subvisual morphometric phenotypes, leading to advancements in patient management [97,99]. Understanding possible risk factors and improving treatment planning for precision oncology may be accomplished by making predictions about patient outcomes based on characteristics or grades generated from histopathologic tumor whole-slide images (WSI) [97]. AI-based computational pathology, in contrast to conventional image-based quantitative analysis, uses a variety of histopathologic image sources and automatic feature-calculation techniques to extract patterns and evaluate characteristics [98]. The development of computer tools and algorithms has made it feasible to estimate cancer patient outcomes using computational pathology, which is essential for modern medicine. However, it is not possible to predict clinical results using pretreatment histopathologic imaging. Huang et al. attempted this when they tested the viability of AI-based algorithms to forecast the effects of neoadjuvant chemotherapy in patients with HER2+ and TNBC using H&E and multiplex IHC (PD-L1, CD8+, and CD163+) images [100]. Although the use of AI in clinical pathology is still in its infancy, this discipline has already demonstrated tremendous promise for enhancing pathology practice in the identification/prediction of clinically actionable biomarkers, such as PD-L1, TILs, and MMR proteins. Several studies have used the WSI of PD-L1 slides and manual supervision to show that image-based scoring algorithms are highly consistent with pathologist reports [101,102,103]. For instance, Wang et al. developed a deep-learning-based AI-assisted model for PD-L1 IC scoring [104]. This study examined the function of the AI-assisted model using 109 PD-L1 (SP142) IHC-stained pictures. The consistency in grading among pathologists might be improved with this method. As a result, the suggested AI-assisted technique may aid pathologists in increasing the accuracy and concordance of the PD-L1 test IC evaluation in breast cancer [104]. In this regard, Sun et al. created a computational TIL evaluation model based on deep learning and assessed the tool’s predictive usefulness for TNBC patients. The authors suggested that pathologists can fulfill risk management and decision-making duties by employing a methodology that incorporates both visual and computational TIL evaluations [105]. They suggest that once validated in larger studies, this algorithm has the potential to serve as a valuable tool for assessing stromal TILs and evaluating prognosis in patients with TNBC. [105]. Moreover, Le et. al. developed and evaluated customized convolutional-neural-network analysis pipelines to generate combined maps of cancer regions and TILs in breast cancer WSI. These combined maps provided insight into the structural patterns and spatial distribution of lymphocytic infiltrates and facilitate improved quantification of TILs [106]. Furthermore, regarding the significant role that MSI/dMMR plays as a biomarker for determining eligibility for immune-checkpoint inhibitors in advanced diseases, some attempts are in progress to predict the MSI/dMMR status through histomorphological features on H&E slides using AI technology [107]. For instance, in the work of Park et al., the authors reported that the model with the highest performance for predicting MSI in colorectal cancer was developed by Lee et al. [108]. In their study, Inception-V3 (a DL-based algorithm) was trained on a cohort composed of images from TCGA and Saint Mary’s Hospital (SMH). When this trained model was tested on an internal validation cohort (TCGA), AUC was 0.892; however, the AUC tested on a different cohort (SMH dataset) was 0.972, which is the highest value reported in the included studies. AI is being increasingly recognized as a promising method for biomarker identification and has subsequently improved clinical management. However, to ensure its successful application, the establishment of robust and standardized computational, clinical, and laboratory practices must be achieved concurrently and validated across multiple collaborating sites. Figure 1 provides an overview of the potential applications of computational pathology in the context of currently available immune-related biomarkers.

## 6. Conclusions

The research on novel therapeutic approaches in TNBC is currently focusing on immunotherapy-specific biomarkers, providing new treatment opportunities for numerous TNBC patients. Although immunotherapy alone has shown limited success in a small subset of TNBC patients, combination strategies are emerging as potential ways to enhance immune responses against tumors. Currently, combining immunotherapy with conventional chemotherapy as a first-line treatment for PD-L1-positive mTNBC has demonstrated significant clinical benefits [109]. Nevertheless, the challenge of treatment resistance, whether inherent from the onset or acquired over time, persists. The tumor microenvironment, where tumor and immune cells interact, plays a crucial role in treatment outcomes. The identification of response-associated biomarkers for ICI is crucial to identify patients who are more likely to have long-lasting responses with minimal side effects. To achieve this, standardized and independently validated assays should be employed in larger prospective studies involving patients with TNBC who are receiving immunotherapy. The identification of reliable prognostic and predictive biomarkers for treatment response is a priority in clinical practice to improve patient selection. 

The improvement of biomarker predictivity will be facilitated by the utilization of advanced technologies capable of providing detailed information about the tumor microenvironment, such as spatial transcriptomics/proteomics and single-cell sequencing [53]. It is probable that additional insights gained from studies on novel biomarkers in different cancer types will contribute to this advancement.

## Figures and Tables

**Table 1:** Comparison of immunohistochemical companion diagnostic assays for PD-L1 assessment in TNBC.

**Assay**	VENTANA PD-L1 (SP142)	PD-L1 IHC 22C3 pharmDx
**Manufacturer**	Roche Diagnostics	Agilent (Dako)
**Scoring system**	IC	CPS
**Cut-off value**	≥1%	≥10
**Evaluation**	Area occupied by PD-L1 stained immune cells (lymphocytes, macrophages, dendritic cells, and granulocytes) as a percentage of the whole tumor area	Summing up PD-L1 stained tumor cells and PD-L1 stained immune cells (lymphocytes and macrophages), divided by the total number of viable tumor cells, and multiplied by 100
**Immune checkpoint inhibitor**	Atezolizumab (Tecentriq©)	Pembrolizumab (Keytruda©)

IC: immune-cell score; CPS: combined positive score.

**Table 2:** Overview of major clinical trials assessing immunotherapy-specific biomarkers in TNBC.

	Study/NCT	Phase	Tumor Type	Drug	Number of Patients	Status
**Observational**	PERCEPTION (NCT04068623)	-	TNBC	-	90	Recruiting
	NCT03165487	-	TNBC	-	30	Recruiting
	TNBCbrazil (NCT03539965)	-	TNBC	-	239	Completed
	NCT05230186	-	Multiple solid tumors	-	200	Recruiting
	TIP (NCT05831553)	-	TNBC	-	100	Recruiting
**Interventional**	TILS001 (NCT05451784)	I/II	Advanced TNBC	NUMARZU-001	20	Not yet recruiting
	Pembro/IORT (NCT02977468)	I	TNBC	Pembrolizumab	15	Recruiting
	NCT04331067	I/II	Localized TNBC	Nivolumab	15	Active, not recruiting
	NCT05556200	II	Early stage TNBC	Camrelizumab	58	Recruiting
	NCT03449108	II	Multiple solid tumors	Autologous tumor infiltrating lymphocytes LN-145	95	Recruiting
	IMpALA (NCT04188119)	II	TNBC	Avelumab	42	Not yet recruiting
	NIB (NCT03289819)	II	TNBC	Pembrolizumab	53	Completed
	START (NCT05492682)	I	Multiple solid tumors	PeptiCRAd-1	15	Recruiting
	NCT03911453	I	TNBC	Rucaparib	20	Active, not recruiting
	ASTEROID (NCT05082259)	I	TNBC	Pembrolizumab	48	Recruiting
	NCT02276443	-	TNBC	Chemotherapy Immunotherapy	1000	Recruiting
	NCT03106415	I/II	Advanced TNBC	Binimetinib	38	Active, not recruiting
	NCT02981303	II	Advanced TNBC	Pembrolizumab	64	Completed
	PAveMenT (NCT04360941)	I	Metastatic TNBC	Palbociclib, Avelumab	45	Recruiting
	NCT05929768	III	Early TNBC	Cyclophosphamide	2400	Not yet recruiting
	NCT03606967	II	Metastatic TNBC	Carboplatin	70	Recruiting
	GeparSixto (NCT01426880)	II/III	Early TNBC	Carboplatin	595	Completed
	ATRC-101 (NCT04244552)	I	Multiple solid tumors	ATRC-101, Pembrolizumab	240	Recruiting

TNBC: triple-negative breast cancer.

## Data Availability

No new data were created.
